# A 24-Year-Old Female Transplant Recipient with Type 2 Membranoproliferative Glomerulonephritis and Disseminated Shingles: A Cautionary Tale of Deferring to Primary Care

**DOI:** 10.1155/2021/6663689

**Published:** 2021-07-24

**Authors:** Benjamin T. Burdorf

**Affiliations:** Northeast Ohio Medical University, Rootstown, OH 44272, USA

## Abstract

In this report, the case of a 24-year-old Caucasian female with type 2 membranoproliferative glomerulonephritis status-post living donor kidney transplant managed on triple regimen immunosuppressive therapy who developed shingles is discussed. With its onset, she promptly reached out to her nephrologist who deferred her to primary care. Prior to seeing her primary provider, she developed disseminated herpes zoster. She consulted emergency services where she was given inadequate care and again deferred to primary care. One day later, the dissemination included her entire torso, face, oral cavity, and all extremities. Fortunately, the patient had the insight to again reach out to her nephrologist who arranged for her to be admitted for appropriate care 6 days after her initial inquiry that carried 6 days of zoster progression. This case demonstrates how it is pertinent that specialists recognize potentially lethal complications associated with the conditions they follow. Although convenient to defer to primary care, if specialists were to take on the responsibility of providing a broader scope of care for their unique subsets of patients, it would likely result in a reduction in the 80% of serious medical errors that occur as a result of miscommunication, or lack thereof, between care providers.

## 1. Introduction

Type 2 membranoproliferative glomerulonephritis (MPGN 2) is a rare disease that affects primarily children and young adults at a prevalence of 2 to 3 individuals per million [1]. Its rarity is coupled with devastation. 50% of patients who incur the illness are subject to end-stage renal disease within 10 years and require dialysis as a bridge to hopeful transplantation [2]. With transplantation, the 5-year graft survival rate among patients with MPGN 2 is approximately 50.0%. It is speculated that this poor outcome is, in part, due to the high incidence of disease recurrence as shown in 67% of posttransplantation biopsies [3]. Current antirejection regimens consist of triple-agent immunosuppressive therapy that includes cyclosporin or tacrolimus, prednisone, and azathioprine or mycophenolate mofetil [4]. Although necessary, life-long immunosuppressive therapy poses a serious concern for the later development of opportunistic infections. Provided the complexity and fragility of these patients, they are inherently followed by multiple providers. As reported by the United States Joint Commission, 80% of serious medical errors occur as a result of miscommunication between care providers [5]. In this report, we will investigate how an episode of shingles eventually disseminated as a result of delayed treatment while being juggled amongst providers.

## 2. Case Report

**Figure 1:**
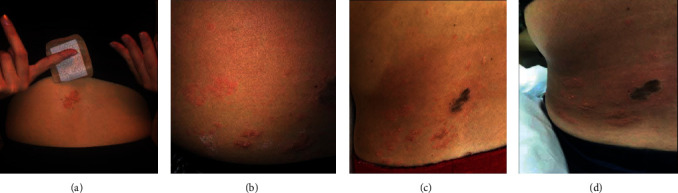
Progression of the initial lesion from left to right. April 16 marks the first day of lesion appearance, April 21 marks her emergency room visit, And April 22 and 23 mark just prior to hospital admission and the following day. (a) April 16, 2020. (b) April 21, 2020. (c) April 22, 2020. (d) April 23, 2020.

**Figure 2:**
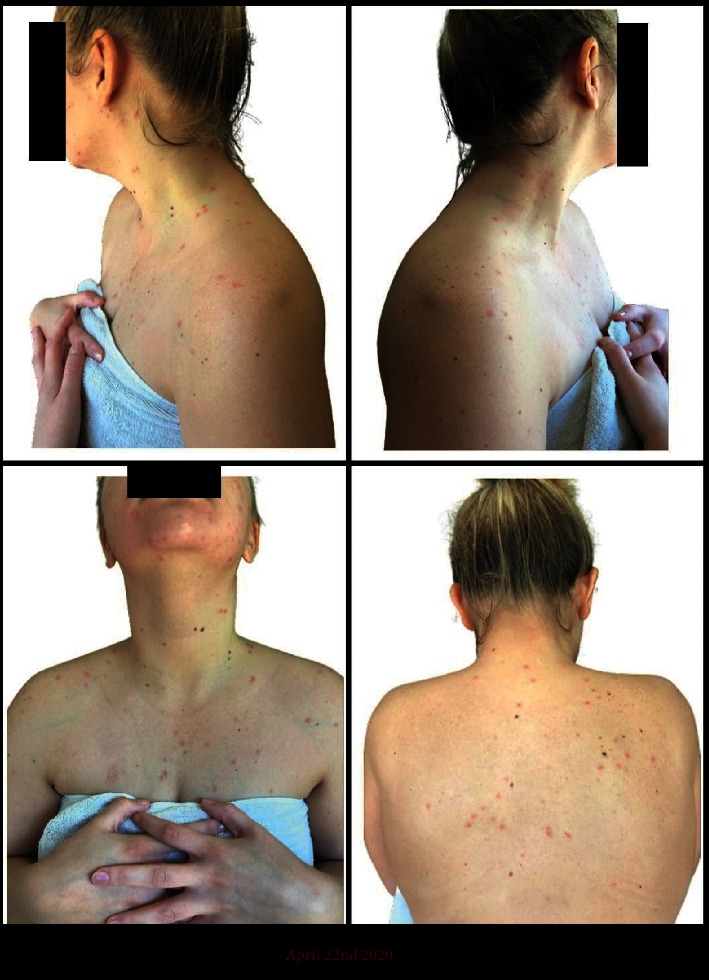
Montage of images portraying the extent of zoster dissemination and progression on April 22, despite having started oral valacyclovir the night prior during her emergency-room visit.

A 24-year-old Caucasian female with a past medical history of hypertension, chronic kidney disease stage 3, and type 2 membranoproliferative glomerulonephritis status-post living donor kidney transplant in 2002 managed on an antirejection regimen of cyclosporin, mycophenolate mofetil, and prednisone noticed the onset of paresthesias and pain in her left lower back the evening of April 13, 2020. The morning of April 16, a grouped vesicular rash positioned along the left 3rd lumbar vertebral dermatome erupted, as shown in Figure 1. Worried about a shingles outbreak, she sent a message that evening to her nephrologist inquiring if this was something that needed to be addressed. The next morning (April 17), she received a response from a care coordinator, on behalf of the nephrologist's office, relaying that she should reach out to her primary-care provider for further work up. Unfortunately, the COVID-19 pandemic meant nonemergent visits were to be conducted via telehealth, an overwhelmed system that had yet to adequately adapt. She was unable to secure an appointment in a timely manner and upon showering the evening of April 21 found her isolated dermatomal rash evolved to include multiple pruritic, vesicular eruptions on her chest. Worried about these developments, the patient reached a nurse on the emergency health services line that informed her she needed to go to the nearest emergency room for suspected disseminated shingles. On arrival, she was afebrile (98.4°F), tachycardic (pulse 114 bpm), and hypertensive (160/107 mmHg). The emergency room physician took the photo shown in Figure 1 labeled April 21 and documented that the patient had similar eruptions in the upper chest and was immune compromised. She was diagnosed with disseminated shingles and sent home with the instructions to again follow-up with her primary doctor and to take valacyclovir 1,000 MG by mouth 3 times per day for 7 days. Despite having started the valacyclovir the night of her emergency room visit, the morning of April 22 showed worrisome progression. The vesicular eruptions that had only been on her left lower back and upper chest became diffusely distributed throughout her torso and now included the neck, face, oral mucosa, and tongue, as shown in Figure 2. Fearful of what may happen if she were to wait for a telehealth appointment with her primary-care provider, she again reached out to her nephrologists relaying her emergency-room course, the plan implemented as well as providing the photos shown in Figure 2. In review of this information, the nephrologist refuted the plan for outpatient oral valacyclovir treatment and coordinated for same day hospital admission. On admission 5 : 00 pm April 22, she was afebrile (98.1°F), tachycardic (pulse 123 bpm), and hypertensive (143/90 mmHg). Laboratory studies showed a bump in her baseline creatinine from ∼1.40 mg/dL to 2.18 mg/dL indicating an acute kidney injury in the setting of viremia, hypertension, and poor oral intake. In addition, the patient's lesions had progressed to include more of her face and had invaded her proximal arms and legs. Fortunately, an ophthalmic exam did not find any involvement of the eyes. PCR analysis was conducted on lesion swabs that were negative for HSV-1 and HSV-2 but positive for varicella zoster confirming the diagnosis of disseminated shingles. She was promptly started on NaCl fluid supplementation and I.V. acyclovir 10 MG/kg twice per day for 5 days. By April 27, the patient's lesions had scabbed over and her creatinine was back to baseline. She was discharged home and instructed to take valacyclovir 1,000 MG twice per day through May 7 at which time she reported a full recovery apart from lesion scarring.

## 3. Discussion

This case demonstrates the importance of timely, prioritized patient management. When the patient initially reached out to her nephrologist, her zoster outbreak was isolated to one dermatome and could have been adequately managed with an outpatient oral antiviral prescription [6]. Instead of addressing the disease at that time, the wait associated with deferment to primary care allowed for the development of viremia and dissemination of the previously contained zoster virus. With dissemination in an immune compromised patient, the virus carries a mortality of 5–15% [7] and inpatient I.V. acyclovir is required as oral antivirals, such as valacyclovir, are no longer sufficient [8]. Furthermore, the delayed treatment of disseminated zoster poses an increased risk for visceral involvement. If the virus reaches this stage, it carries a more ominous outlook with an estimated mortality of 55% [9]. Fortunately, this patient had the awareness to seek care from emergency services at the first sign of dissemination. Unfortunately, she was given inadequate treatment (outpatient management with oral valacyclovir) and again deferred to primary care. As discussed, her condition continued to deteriorate as the virus wreaked havoc, manifesting within her torso, face, oral cavity, and all extremities just one day after her emergency-room visit. Thankfully, the patient again reached out to her nephrologist who took the action to have her admitted to finally receive the standard of care 6 days after her initial inquiry that carried 6 days of zoster progression. It is regrettable that, in this case, the initiative and insight to obtain adequate care continued to fall on the patient. While shingles is usually managed through primary care and the emergency-room physician provided treatment that is sufficient in most settings, a solid organ transplant recipient with recurrent type 2 membranoproliferative glomerulonephritis on triple immunosuppressive therapy falls within a unique subset of patients that often require different standards of management. Because triple immunosuppressive therapy is the standard of care, special attention should be provided to prophylactic measures for opportunistic infections. As of 2019, it is recommended that kidney transplant recipients who are varicella zoster virus seropositive receive the inactivated recombinant subunit herpes zoster vaccine, which would have likely avoided this incident entirely had it been given [10]. Provided the complexity of patients followed in an interdisciplinary manner, it is pertinent that specialists recognize potentially lethal complications associated with the conditions they follow. Although convenient to defer to primary care, if specialists were to take on the responsibility of providing a broader scope of care for their unique subsets of patients, it would likely result in a reduction in the 80% of serious medical errors that occur as a result of miscommunication, or lack thereof, between care providers.

## Data Availability

The data used to support the findings of this study are included within the article (in references).
